# BEYOND REPRODUCTION: EXPLORING DETERMINANTS OF FUNCTIONAL HEALTH AND WELL-BEING IN AGING WOMEN

**DOI:** 10.1093/geroni/igz038.2252

**Published:** 2019-11-08

**Authors:** Cassandra M Germain

**Affiliations:** North Carolina A&T State University, Greensboro, North Carolina, United States

## Abstract

The goal of this symposium is to explore the various factors that influence functional health and general well-being in women during late life. We will explore 1) the role of depression on functional impairment in a group of diverse community dwelling women; 2) the relationship between chronic pain and depression, as well as strategies for intervention; 3) the desire for sexual intimacy among older women and it’s relationship to wellbeing and 4) the importance of physical appearance in older women and its association with self confidence and wellbeing.

